# Echocardiographic assessment of subclinical left ventricular eccentric hypertrophy in adult-onset GHD patients by geometric remodeling: an observational case-control study

**DOI:** 10.1186/1472-6823-6-1

**Published:** 2006-02-28

**Authors:** Cesare de Gregorio, Lorenzo Curtò, Antonino Recupero, Patrizia Grimaldi, Barbara Almoto, Marilena Venturino, Domenico Cento, Maria Carola Narbone, Francesco Trimarchi, Sebastiano Coglitore, Salvatore Cannavò

**Affiliations:** 1 Clinical and Experimental Department of Medicine and Pharmacology, Cardiology Unit, University Hospital of Messina, Messina, Italy; 2 Clinical and Experimental Department of Medicine and Pharmacology, Endocrine Unit, University Hospital of Messina, Messina, Italy; 3 Department of Neurosciences, University Hospital of Messina, Messina, Italy

## Abstract

**Background:**

Most patients with growth hormone deficiency (GHD) show high body mass index. Overweight subjects, but GHD patients, were demonstrated to have high left ventricular mass index (LVMi) and abnormal LV geometric remodeling. We sought to study these characteristics in a group of GHD patients, in an attempt to establish the BMI-independent role of GHD.

**Methods:**

Fifty-four patients, 28 F and 26 M, aged 45.9 ± 13.1, with adult-onset GHD (pituitary adenomas 48.2%, empty sella 27.8%, pituitary inflammation 5.5%, cranio-pharyngioma 3.7%, not identified pathogenesis 14.8%) were enrolled. To minimize any possible interferences of BMI on the aim of this study, the control group included 20 age- and weight-matched healthy subjects. The LV geometry was identified by the relationship between LVMi (cut-off 125 g/m^2^) and relative wall thickness (cut-off 0.45) at echocardiography.

**Results:**

There was no significant between-group difference in resting cardiac morphology and function, nor when considering age-related discrepancy. The majority of patients had normal-low LVM/LVMi, but about one fourth of them showed higher values. These findings correlated to relatively high circulating IGF-1 and systolic blood pressure at rest. The main LV geometric pattern was *eccentric hypertrophy *in 22% of GHD population (26% of with severe GHD) and in 15% of controls (p = NS).

**Conclusion:**

Though the lack of significant differences in resting LV morphology and function, about 25% of GHD patients showed high LVMi (consisting of *eccentric hypertrophy*), not dissimilarly to overweight controls. This finding, which prognostic role is well known in obese and hypertensive patients, is worthy to be investigated in GHD patients through wider controlled trials.

## Background

Growth hormone (GH) is essential for the regulation of body composition, nutrient metabolism, extra-cellular fluid volume, lean and fat mass, maintenance of muscle mass and strength, myocardial structure and function [1-5].

In the majority of the patients with adult-onset and/or congenital growth hormone deficiency (GHD), previous studies showed the presence of cardiac abnormalities, mainly consistent with reduced left ventricular (LV) wall thickness, myocardial hypotrophy, impaired LV performance at rest or exertion, often associated with obesity. Some of these findings have been related to increased cardiovascular risk [6-18].

However, there is only scant information about GHD patients who have higher LV mass (LVM). Previous clinical trials clearly demonstrated that in obese and hypertensive patients with high LVM, increased relative risk for cardiovascular events is related to abnormal LV geometric remodeling [19-23].

Given that most GHD patients show high body mass index (BMI), we managed to study the echocardiographic characteristics of LVM and LV remodeling on this account. In order to establish whether there is a BMI-independent role of GHD on cardiac mass, the study group was compared to an age- and weight-matched control group.

## Methods

### Study population

Fifty-four consecutive Caucasian patients, 28 women and 26 men, aged 45.9 ± 13.1 (range 19–67), with adult-onset GHD were enrolled from January 2000 to May 2005. The main diagnosis in these patients was pituitary adenomas in 26 (48.2%), empty sella in 15 (27.8%), previous pituitary inflammation in 3 (5.5%), cranio-pharyngioma in 2 (3.7%). The pathogenesis was not identified in 8 patients (14.8%).

Isolated GHD was observed in 16 patients (29.6%). Of the 31 patients with adenomas, inflammation or cranio-pharyngioma, 25 had undergone surgery and 10 also received radiotherapy.

The diagnosis of GHD was based on the decreased GH responsiveness (GH-peak) to pyridostigmine + GH releasing-hormone stimulation (oral administration of pyridostigmine 120 mg, followed, after 60 minutes, by GH-RH 100 ng/mL iv) and circulating IGF-I levels [24].

The GHD patients were divided into 2 groups based on the response to test : GH-peak was < 3 ng/mL in 38 patients (group A, severe GHD), and 3 to 17 ng/mL in 16 (group B, mild GHD).

Circulating IGF-1 was also tested in the GHD population. The normality range had been previously established in our Institution as follows: a) 131–384 ng/mL in patients aged 18 to 35, b) 100–312 ng/mL in those aged 36 to 50, and c) 106–270 ng/mL in over 50s.

Free triiodothyronine (FT3) and thyroxine (FT4) were measured in all the study population.

The onset of GHD (GHD length) was identified on the basis of clinical history (first diagnosis of pituitary disease, prior surgery, and, if any, previous chemical assays).

On admission, each patient underwent clinical evaluation, including measurements of weight, height, body mass index (BMI), sitting blood pressure, heart rate, standard 12-lead electrocardiography and Doppler echocardiography.

Acute coronary syndrome, previous myocardial infarction, congestive heart failure, congenital heart disease, ventricular arrhythmias, asthma, cancer, severe renal and hepatic dysfunction were all considered as exclusion criteria.

The assessment of high blood pressure was carried out in accordance with the current guidelines [25]. Patients who showed mild-to-moderate hypertension were included in the study. The final study group was compared to an age- and weight-matched control group of 20 subjects with no history of cardiac disease, from the same geographic region.

### Cardiac ultrasound

Transthoracic Doppler echocardiography was performed with a commercial ultrasound unit equipped with 2.5–3.5 MHz transducers in harmonic imaging. Patients were examined in the left lateral supine decubitus after 15 minutes resting by an experienced physician, and data stored on magneto-optical disks.

Left ventricular end-diastolic/end-systolic diameters, interventricular septum (IVS) and posterior wall (PW) thickness, absolute and indexed LV mass (LVM and LVMi), relative wall thickness (RWT = IVS + PW thickness/LV end-diastolic diameter), endocardial and midwall fractional shortening (EFS and MFS) were measured according to recommendations of the American Society of Echocardiography and other studies [21,22,26,27].

Based on the relationship between LVMi and RWT, using the cut-off values of 125 g/m^2 ^for LVMi and 0.45 for RWT, both in men and women, the following geometric models were identified: *normal geometry*, for RWT≤0.45 and LVMi≤ 125 g/m^2^; *concentric remodeling*, for RWT>0.45 and LVMi≤125 g/m^2^; *eccentric hypertrophy*, for RWT≤0.45 and LVMi>125 g/m^2^; and *concentric hypertrophy*, for RWT>0.45 and LVMi>125 g/m^2 ^[20-23].

The general distribution of LVM and LVMi in the GHD population was also recognised, and 3 different categories based on 2 median values were identified.

Once no wall motion abnormalities were found, LV ejection fraction was calculated by the single-plane Simpson rule method (LV diastolic volume – LV systolic volume/LV diastolic volume) from the 4-chamber apical view.

Diastolic function was evaluated by Doppler sampling at LV inflow [trans-mitral valve blood flow sampling: early (E) and late (A) peak velocity, E/A ratio, E-wave deceleration time] and the upper right pulmonary vein outflow [systolic (S) and diastolic (D) velocity, S/D ratio, and reverse atrial velocity (Ap)], in accordance with the current European guidelines [28].

### Statistical analysis

Continuous variables are expressed as mean ± SD, except for data expressed in percents (%). Exact Fisher test, analysis of variance with either Scheffé, Kruskal-Wallis, or chi-squared test when appropriate, were used to check the between-group differences.

Subjects' median age identified the age-related differences in LVM and LV systolic/diastolic functional parameters, and the differences were checked out by Student-T test. Linear correlation between LVMi and IGF-I and GH-peak was performed and a multivariate analysis was done in order to establish the main determinants of LVMi in the whole GHD population. The null hypothesis was rejected at 2 tails for p < 0.05 (95% CI).

## Results

### Clinical features

Demographic and clinical characteristics of the study population are displayed in Table 1. With the exception of GH-peak and circulating IGF-1 (both lower in group A than in B, p < 0.0001 and < 0.05, respectively), there were no significant differences in basal values.

**Table 1:** Demographic and clinical characteristics of the study population.

	Severe GHD (n = 38)	Mild GHD (n = 16)	Controls (n = 20)	p-value
Age	44.9 ± 14.0	48.2 ± 10.6	45.5 ± 12.0	NS
Gender (M/F)	23/15	3/11	9/11	NS
NYHA class	1.3 ± 0.5	1.1 ± 0.1	1.2 ± 0.1	NS
Body mass index	29.7 ± 6.8	31.4 ± 4.8	28.6 ± 4.4	NS
SBP (mmHg)	134.1 ± 16.1	133.6 ± 17.6	128.1 ± 10.3	NS
DBP (mmHg)	79.5 ± 10.8	81.8 ± 9.8	78.5 ± 11.1	NS
SBP > 135 and/or DBP > 85 mmHg	11 (28.4)	6 (37.5)	5 (25%)	NS
GH-peak (ng/mL)	0.97 ± 0.84	8.31 ± 3.31	NA	<0.0001
IGF-1 (ng/mL)	94.3 ± 64.6	130.5 ± 65.8	NA	<0.05
FT3 (mmol/L)	4.2 ± 1.2	4.9 ± 1.6	5.3 ± 0.9	NS
FT4 (mmol/L)	12.9 ± 4.0	15.5 ± 3.8	14.2 ± 2.9	NS

Values are mean ± SD.

*Legends: DBP*, Diastolic Blood Pressure; *GH*, Growth Hormone; *IGF-1*, Insulin-Like Growth Factor; *NA*, Not Applicable; *NYHA*, New York Heart Association Functional Class; *SBP*, Systolic Blood Pressure; *FT3*, Free Triiodothyronine; *FT4*, Free Thyroxine.

A higher, but not significant, number of females was present in group B (68.7%) than in A (39.5%) and C (55%).

Growth hormone deficiency length was established from 8 to 384 months (mean value 154 ± 115 months).

At entry, some patients were already treated with specific substitutive hormone for hypothyroidism (n = 29, 53.7%), hypogonadism (n = 26, 48.1%), hyposurrenalism (n = 25, 46.3%) and diabetes insipidus (n = 5, 9.2%), alone or in combination. Thus, FT3 and FT4 serum levels were comparable among the 3 groups.

### Cardiac morphology and function

Standard 12-lead ECG showed normal sinus rhythm in each patient, with no evidence of significant arrhythmias (atrial fibrillation, atrial flutter, premature ventricular beats >100/h, non-sustained tachycardia) and/or ST-T wave abnormalities suggestive of coronary artery disease.

Basal echocardiographic findings are displayed in Table 2. The main difference regarded the higher prevalence of extra-pericardial fat deposit in GHD patients than in controls (p < 0.001).

**Table 2:** Resting echocardiographic measurements

	Severe GHD (n = 38)	Mild GHD (n = 16)	Controls (n = 20)	p-value
***M-mode and two-dimensional parameters***				
LVEDD (mm)	49.4 ± 5.8	49.1 ± 4.0	49.3 ± 4.0	NS
LVESD (mm)	30.7 ± 4.5	29.4 ± 3.9	30.1 ± 3.5	NS
IVS (mm)	9.8 ± 1.8	10.6 ± 1.4	10.0 ± 1.5	NS
PW (mm)	7.8 ± 1.4	7.8 ± 1.3	8.4 ± 1.4	NS
RWT	0.36 ± 0.05	0.38 ± 0.05	0.38 ± 0.06	NS
LVM (g)	194.2 ± 62.6	197.8 ± 46.6	195.3 ± 44.5	NS
LVMi (g/m^2^)	104.8 ± 27.6	109.1 ± 24.9	106.1 ± 22.7	NS
LVDV (ml)	82.3 ± 20.7	77.2 ± 19.2	80.8 ± 15.5	NS
LVSV (ml)	30.4 ± 9.2	27.4 ± 8.3	31.7 ± 6.6	NS
LA systolic area (cm^2^)	16.8 ± 3.8	15.7 ± 2.5	16.7 ± 1.4	NS
RA systolic area (cm^2^)	14.2 ± 2.9	13.6 ± 2.2	14.1 ± 1.3	NS
Pericardial adiposity	26 (68.4%)*	10 (71.4%)*	4 (20%)	<0.001

***Left ventricular function***				
EFS (%)	39.0 ± 5.1	40.1 ± 5.5	38.8 ± 4.9	NS
MFS (%)	18.6 ± 2.7	18.8 ± 1.8	18.1 ± 2.3	NS
LVEF (%)	63.5 ± 5.8	63.6 ± 9.0	61.5 ± 5.3	NS
Mitral E/A velocity ratio	1.24 ± 0.56	0.99 ± 0.27	1.18 ± 0.48	NS
E-dt	175.5 ± 36.8	195.7 ± 53.2	173.7 ± 39.8	NS
PV S/D ratio	1.1 ± 0.6	1.0 ± 0.5	1.3 ± 0.3	NS
PV A velocity	28.9 ± 3.2	29.3 ± 5.2	27.3 ± 4.2	NS

Values are mean ± SD. Scheffé test for comparison of individual groups: *p < 0.01 (groups A-B vs C).

*Legends: E/A*, Early/late diastolic velocity at left ventricular (LV) inflow; *E-dt*, E-velocity deceleration time; *EFS*, Endocardial fractional shortening; *IVS*, Interventricular septal thickness; *LA*, Left atrium; *LVDV*, LV diastolic volume; *LVEDD*, LV end-systolic diameter; *LVEF*, LV ejection fraction; *LVESD*, LV end-systolic diameter; *LVM/LVMi*, absolute/indexed LV mass; *LVSV*, LV systolic volume; *MFS*, midwall fractional shortening; *PV*, Right superior pulmonary vein; *PW*, Posterior wall thickness; *RA*, Right atrium; *RWT*, Relative wall thickness; *S/D*, Systolic/Diastolic velocity.

**Figure 1:**
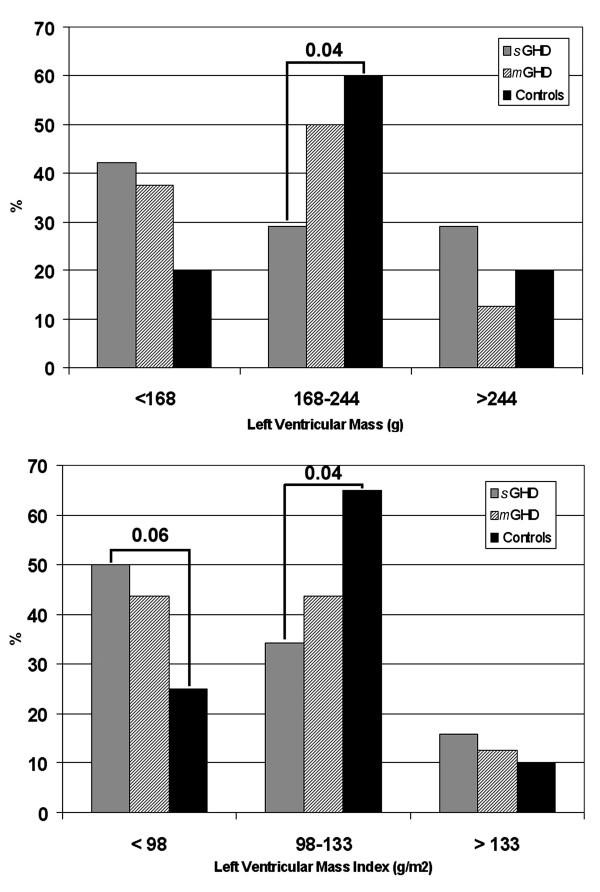
Prevalence of left ventricular mass and left ventricular mass index in the study population. LEGEND:*m-*GHD, patients with mild GHD; *s-*GHD, patients with severe GHD.

Average values of LVM/LVMi were comparable among the groups. Figure 1 also shows the median-related distribution of LVM and LVMi in 3 categories (LVM <168 g, 168–244 g, >244 g, and LVMi <98 g/m^2^, 98–133 g/m^2^, >133 g/m^2^) for each study group. More than 70% of patients with severe GHD had LVM < 244 g and LVMi < 133 g/m^2^.

**Figure 2:**
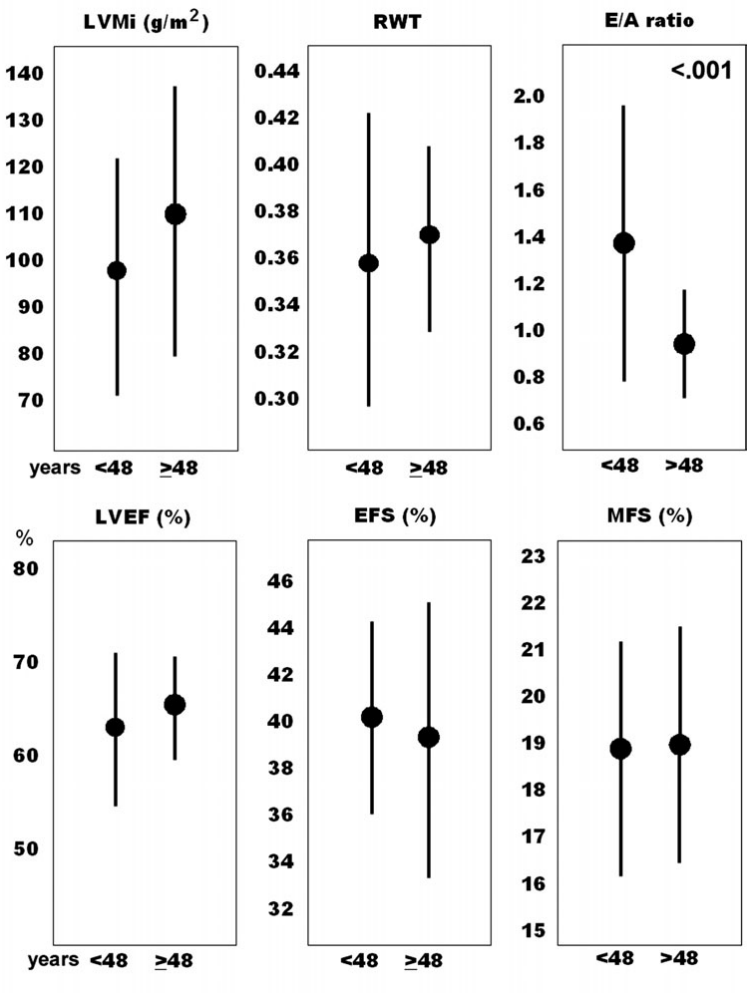
Age-related differences in left ventricular mass index (LVMi), relative wall thickening (RWT), mitral E/A velocity ratio (E/A ratio), left ventricular ejection fraction (LVEF), endocardial fractional shortening (EFS), and midwall fractional shortening (MFS), in the GHD population (n = 54).

No significant age-related difference in LVMi, RWT, and systolic functional parameters was observed within the GHD group (Figure 2). Only a decrease in mitral E/A ratio was consistent with age, the same as reported in the general population [28].

**Figure 3:**
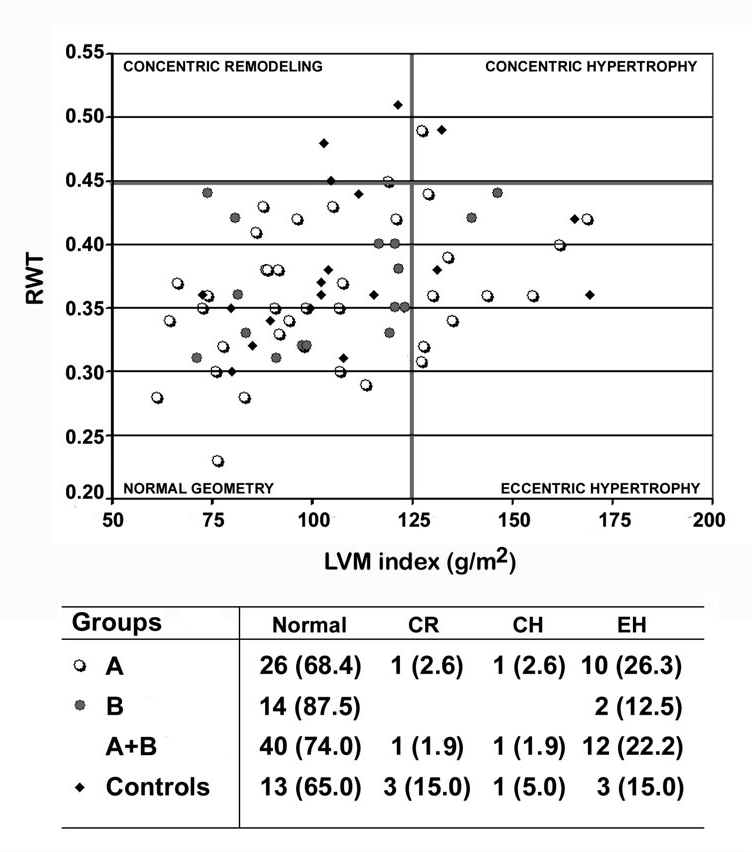
Scatter plot illustrating the left ventricular remodeling in severe (open circles), partial (full circles) GHD and controls (squares). Numbers and percents (%) of the cases in each group are displayed in the table underneath. Legends: *CR*, concentric remodeling; *CH*, concentric hypertrophy; *EH*, eccentric hypertrophy; *LVMi*, left ventricular mass index; *RWT*, relative wall thickness.

Analysis of the LV geometric remodeling showed that the majority of GHD patients and controls had "*normal geometry*". Twelve GHD patients (10 with severe deficiency) showed "*eccentric hypertrophy*" (22.2% vs 15.0% in controls, NS). One patient from group A had "*concentric remodeling*" and another from the same group had "*concentric hypertrophy" *(Figure 3).

Four out of the 12 GHD patients with "*eccentric hypertrophy*" (33.3%), the patient with "*concentric remodeling*" and 1/3 of controls with "*eccentric hypertrophy*" (33.3%) suffered from systemic hypertension (NS).

However, in comparison with the GHD patients with low-normal LVM/LVMi (n = 41), those with high cardiac mass (n = 13) showed greater systolic blood pressure (131.7 ± 16.3 vs 118.2 ± 16.9 mmHg in the former group, respectively; p = 0.02) and diastolic blood pressure (81.3 ± 7.1 vs 74.8 ± 11.0 mmHg, respectively; p = 0.06), measured on admission.

**Figure 4:**
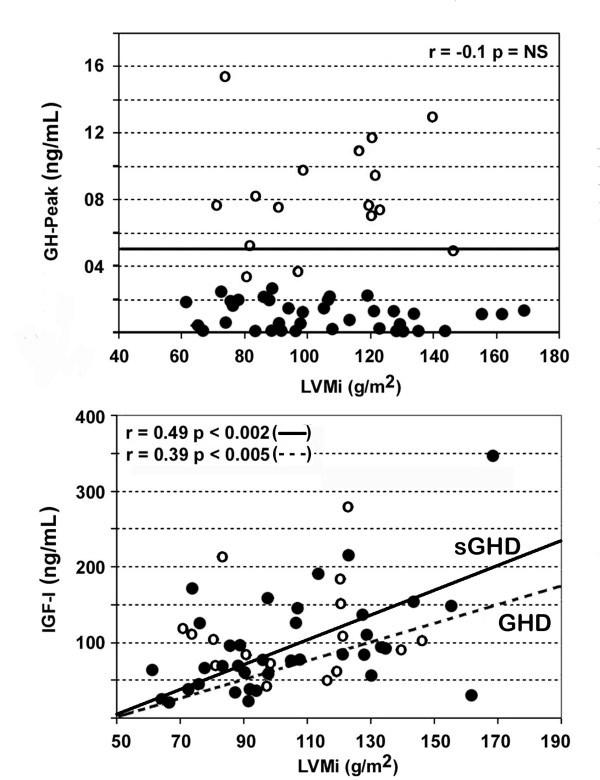
Linear relationship between circulating IGF-1 and left ventricular mass index (LVMi) in patients with mild (open circles) and severe (full circles) GHD. Correlation r-values and p-values in both groups and in patients with severe GHD are reported.

Overall, there was a moderate, but significant, correlation between LVMi and circulating IGF-I in the whole GHD population (r 0.39, p < 0.005), and particularly in group A (r 0.49, p < 0.002) (Figure 4). And IGF-1 was confirmed to be the main determinant for LVMi at multivariate analysis (Table 3).

**Table 3:** *Main determinants of LVMi in the GHD patient group at multivariate analysis*.

**Variable**	**Regression (B)**	**SE**	**t-value**	**p-value**
Age	0.3090	0.3756	0.8229	0.416
GHD length	0.0056	0.0377	0.1485	0.883
GH-peak	-0.4494	0.9574	0.4693	0.642
IGF-1	0.2646	0.0056	2.7553	0.009*
SBP	0.3869	0.3063	1.2633	0.214

*Constant (C) = 29.193. Determinant coefficient (r squared) = 0.27*.

*Legends: GH*, growth hormone; *IGF-1*, insulin-like growth factor; *SE, s*tandard error; *SBP*, systolic blood pressure.

Colour-flow mapping and Doppler sampling allowed identification of trivial mitral valve regurgitation in 31 patients from group A (81.6%), 13 from group B (92.8%), and 17 from group C (85%) (NS).

## Discussion

The main findings from the present study indicate that there is no significant difference in left ventricular morphology and resting function between adult-onset GHD patients and overweight healthy subjects.

As already demonstrated in other GHD populations, about 75% of our patients had low or normal LVM/LVMi [2-11]. Conversely, about a quarter of them had increased values.

We know that high LVMi emerged as the most important prognostic determinant for cardiovascular events in patients with obesity and or hypertension [19-22,27,29]. While analysing the LV geometric remodeling, it was established that LV *concentric hypertrophy *has 2.1–3.6 annual odds ratio, eccentric hypertrophy 1.0–2.9, and concentric remodeling 0.3–2.4, for negative outcomes [20-22,29].

Based on previous literature data on BMI in GHD patients, we managed to evaluate whether the analysis of LV geometric remodeling could improve the identification of those subgroups at risk for cardiovascular events, in relation with the higher LVMi. To have found LV *eccentric hypertrophy *in 22% of the cases (26% of with severe GHD) likely implies that some patients are, from this point of view, comparable to obese individuals, where this pattern usually occurs in more than 20% of cases. Given the specifc risk rate recognised in these latter category of patients, we may assume that this minority of GHD patients who show LV hypertrophy deserves further attention due to an equivalent estimated risk [19-22,29].

On the other hand, *eccentric hypertrophy *has been regarded as an effective way to keep systolic function into normality in obese patients, by resorting to the Starling reserve [22,23]. This adaptive mechanism is likely to play a role even in GHD patients, were we found no resting LV (systolic and diastolic) dysfunction in comparison to controls.

Hence, our results are in agreement with Ozbey et al, who demonstrated normal cardiac dimensions and LV systolic function at rest in the majority of their GHD patients [30].

On the contrary, depressed systolic function at rest with abnormal exertion response were recently shown by Colao et al in about 79% of patients with severe GHD, 44% with mild GHD, and 6% of controls [31].

In our opinion, such conflicting results between previous and present findings might be due to patients' age, length of GHD and extent of pituitary disease, methods to assess LV morphology and function, and local phenotypic characteristics as well [32-35].

The main limitations of this study is the inadequate patients' number to draw definite conclusions and the lack of information about LV performance at exercise. However, to evaluate the LV function was not a primary end-point. In fact, cardiovascular function of GHD patients, both at rest and/or exertion, can be affected by several co-morbidities, as coronary artery disease (even clinically silent), systemic hypertension, diabetes, metabolic syndrome, lung disease, multiple endocrine dysfunction, which should be all adequately screened [31-36].

Beyond greater circulating IGF-1, our patients with LV hypertrophy also showed slight increase in blood pressure at baseline, which pathophysiologic role on the modulation of cardiac mass is well known [21].

The Paris prospective study, a large controlled trial where the independent prognostic role of GH was investigated in the general population, clearly demonstrated that cardiovascular disorders mainly correlate to GH increase rather than to deficiency [37]. Accordingly, it has been unquestionably established that acromegalic patients are at high risk for cardiovascular events, due to development of LV hypertrophy, high blood pressure, and early vascular atherosclerosis [4,7,15,37-39].

## Conclusion

Findings from the present study show that LV morphology and resting function are not significantly different between GHD patients and age- and weight-matched control subjects.

Overall, these patients had low-normal LVM and LVMi, and high prevalence of pericardial fat deposit. However, in about 22% of them (26% of with severe GHD) an increase in LVMi, similar to overweight controls, can be observed. The main geometric pattern consists of LV eccentric hypertrophy.

In this series, LVMi was found to correlate with relatively high circulating IGF-1, but not to GH-peak or GHD length, and with resting systolic blood pressure.

Therefore, the analysis of the LV geometric remodeling appears to be such a simple echocardiographic method that can help physicians to better identify which category of GHD patients is likely to be at risk for cardiovascular events, strictly due to changes in cardiac mass.

Further study is needed to validate our results and establish their actual prognostic impact.

## Abbreviations

CH – Concentric Hypertrophy

CR – Concentric Remodeling

DBP – Diastolic Blood Pressure

EFS – Endocardial Fractional Shortening

EH – Eccentric Hypertrophy

GHD – Growth Hormone Deficiency

IGF-1 – Insulin-like Growth Factor-1

LV – Left Ventricle/Ventricular

LVM – Left Ventricular Mass

LVMi – LVM index (normalized to body surface area)

MFS – Midwall Fractional Shortening

OR – Odds Ratio

RWT – Relative Wall Thickness

SBP – Systolic Blood Pressure

## Competing interests

The author(s) declare that they have no competing interests.

## Authors' contributions

The project idea was conceived by Dr Cesare de Gregorio, who also collected and analysed the echocardiographic data, performed statistical analyses, drafted and revised the manuscript. All co-authors gave contribution to the manuscript and made suggestions for revisions.

Endocrine data were collected and edited by Dr Salvatore Cannavò, Dr Lorenzo Curtò, Dr Barbara Almoto, and Dr Marilena Venturino. Echocardiographic data were also collected by Dr Antonino Recupero and Dr Patrizia Grimaldi. Neurological evaluation was performed by Dr Maria Carola Narbone.

## Appendix

Preliminary results from the study were presented as brief communications at the 5^th ^European Congress of Endocrinology, Turin (Italy), 2001 June 9–13.

## Pre-publication history

The pre-publication history for this paper can be accessed here:


